# Cardiometabolic risk factors, physical activity, and postmenopausal breast cancer mortality: results from the Women’s Health Initiative

**DOI:** 10.1186/s12905-022-01614-3

**Published:** 2022-02-05

**Authors:** Christina M. Dieli-Conwright, Rebecca A. Nelson, Michael S. Simon, Melinda L. Irwin, Marian L. Neuhouser, Kerryn W. Reding, Tracy E. Crane, JoAnn E. Manson, Rami Nassir, Aladdin H. Shadyab, Michael LaMonte, Lihing Qi, Cynthia A. Thomson, Candyce H. Kroenke, Kathy Pan, Rowan T. Chlebowski, Joanne Mortimer

**Affiliations:** 1 Division of Population Sciences, Dana-Farber Cancer Institute, 375 Longwood Ave, Boston, MA 02215 USA; 2 Harvard Medical School, Boston, MA USA; 3 City of Hope National Medical Center, Duarte, CA USA; 4 Karmanos Cancer Institute, Detroit, MI USA; 5 Yale University, New Haven, CT USA; 6 Fred Hutchinson Cancer Research Center, Seattle, WA USA; 7 University of Washington, Seattle, WA USA; 8 University of Miami, Coral Gables, FL USA; 9 Umm Al-Qura’a University, Mecca, Saudi Arabia; 10 University of California, San Diego, San Diego, CA USA; 11 University of California, Davis, Davis, CA USA; 12 University of Arizona, Tucson, AZ USA; 13 Kaiser Permanente Northern California Division of Research, Oakland, CA USA; 14 Lundquist Institute for Biomedical Innovation at Harbor-UCLA Medical Center, Torrance, CA USA

**Keywords:** Physical activity, Metabolic syndrome, Breast cancer

## Abstract

**Background:**

Higher physical activity levels are associated with lower breast cancer-specific mortality. In addition, the metabolic syndrome is associated with higher breast cancer-specific mortality. Whether the physical activity association with breast cancer mortality is modified by number of metabolic syndrome components (cardiometabolic risk factors) in postmenopausal women with early-stage breast cancer remains unknown.

**Methods:**

Cardiovascular risk factors included high waist circumference, hypertension, high cholesterol, and diabetes. Breast cancers were verified by medical record review. Mortality finding were enhanced by serial National Death Index queries. Cox proportional hazards regression models were used to estimate associations between baseline physical activity and subsequent breast cancer-specific and overall mortality following breast cancer diagnosis in Women’s Health Initiative participants. These associations were examined after stratifying by cardiometabolic risk factor group.

**Results:**

Among 161,308 Women’s Health Initiative (WHI) participants, 8543 breast cancers occurred after 9.5 years (median) follow-up in women, additionally with information on cardiometabolic risk factors and physical activity at entry. In multi-variable analyses, as measured from cancer diagnosis, higher physical activity levels were associated with lower all-cause mortality risk (hazard ratio [HR] 0.86, 95% confidence interval [CI] 0.78–0.95, trend *P* < 0.001) but not with breast cancer-specific mortality (HR 0.85, 95% CI 0.70 to 1.04, trend *P* = 0.09). The physical activity and all-cause mortality association was not significantly modified by cardiometabolic risk factor number.

**Conclusions:**

Among women with early-stage breast cancer, although higher antecedent physical activity was associated with lower risk of all-cause mortality, the association did not differ by cardiometabolic risk factor number.

## Background

Metabolic syndrome is a clustering of metabolic dysfunctions that includes at least three of the following: high waist circumference, triglycerides, blood pressure, and fasting blood glucose; and lower high-density lipoprotein cholesterol. Among other associated morbidities, the metabolic syndrome has been associated with higher risk of breast cancer-specific mortality [1, 2].

Higher physical activity levels, compared to lower levels, measured prior to breast cancer diagnosis have been associated with statistically significantly lower all-cause mortality among women diagnosed with breast cancer [3, 4]. Higher physical activity levels have been shown to influence the metabolic syndrome in women with breast cancer [5] and in other settings [6] where exercise programs significantly reduced metabolic syndrome components. However, it is unknown whether presence of the metabolic syndrome modifies the favorable physical activity influence on breast cancer. Therefore, we examined the association between physical activity and all-cause mortality after breast cancer and breast cancer-specific mortality to determine whether associations were modified by the number of metabolic syndrome components. We hypothesized that women with breast cancer having more metabolic syndrome components would be more likely to benefit from higher pre-diagnosis physical activity levels in terms of breast cancer mortality.

## Methods

The Women’s Health Initiative (WHI) recruited 161,308 post-menopausal women aged 50–79 with anticipated 3-year survival from 40 US clinical centers between 1993 through 1998. Details of the WHI have been reported [7]. All protocols were approved by institutional review boards and participants gave written informed consent. Eligible for the current analyses were participants with incident, invasive, non-metastatic breast cancer (n = 10,124), additionaly with baseline measurement of cardiometabolic risk factors (n = 8543).

Participant characteristics were collected using questionnaires at entry regarding breast cancer risk factors, demographics, physical activity, medical history, and social economic status. Blood pressure was measured using standardized procedures by certified personnel with two determinations taken 30 s apart and averaged. Waist circumference, weight, and height were measured by trained personnel using a standardized approach with body mass index (BMI) computed as weight (kg)/[height (m)]^2^.

Physical activity was assessed by questionnaire. Participants were asked about walking outside the home by duration and by speed categories; metabolic equivalent task (MET) values were assigned for walking (average, 3 METs; fast, 4 METs; and very fast 4.5 METs). For recreational physical activity, participants were asked how often and for how long they exercised at various intensity levels. Vigorous-intensity activities included aerobics, jogging, tennis, and swimming laps while moderate-intensity activities included biking, exercise machine use, calisthenics, easy swimming, and dancing. The information on walking and recreational physical activity was used to generate a hours/week (h/wk) variable which was combined with MET values for each activity.to generate the final MET-h/wk variable, as previously described [8].

A convenience construct was used to assess metabolic syndrome components, referred to here as “cardiometabolic risk factors” which were determined at entry and included: (1) measured high waist circumference, (2) measured high blood pressure, (3) history of high cholesterol, and (4) history of diabetes, as previously described [4]. Information on triglyceride levels was not available. High blood pressure was defined as systolic ≥ 130 mmHg and/or diastolic ≥ 85 mmHg, or a normal blood pressure and use of anti-hypertensive medications identified during in-clinic medication inventory. High waist circumference was defined as ≥ 88 cm [8]. High cholesterol was determined using the question “Has a doctor ever told you that you had high cholesterol requiring medication?” or cholesterol-lowering medication use identified during in-clinic medication inventory. Diabetes was determined using the question “Did a doctor ever say that you had sugar diabetes or high blood sugar when you were not pregnant?” or by use of diabetes-related medication use identified during in-clinic medication inventory. This diabetes definition has been validated and is consistent with medication inventories [9].

In the WHI clinical trials, participants were followed for clinical outcomes every 6 months during the 8.5-year (median) intervention period, then annually. WHI observational study participants were followed annually throughout. Breast cancers were initially confirmed with medical record review at the clinical centers by trained physician adjudicators with final confirmation and coding at the clinical coordinating center. Reports of death and cause of death were verified by medical records or death certificate review at the clinical coordinating center [10]. Mortality findings were enhanced by serial National Death Index queries resulting in survival information which was 98% complete [11]. Breast cancer therapy was directed by participant’s own physicians. Current study outcomes of interest included all-cause mortality and breast cancer-specific mortality, both measured from breast cancer diagnosis.

### Statistical analysis

Cardiometabolic risk groups were categorized into 0, 1–2, and 3–4 components as incorporated in previous analyses [12]. Associations of physical activity with all-cause mortality after breast cancer and breast-cancer specific mortality were assessed using multivariable Cox proportional hazards regression models and trend tests for the ordinal variable. Mortality outcomes were measured from breast cancer diagnosis. Multivariable models were adjusted for confounders found significant (adjusts for age group, race/ethnicity alcohol intake, smoking status, BMI kg/m^2^, # of metabolic risk factors, hormone receptor status, grade, HER2/NEU number of examined lymph nodes, number of positive lymph nodes, tumor size, and stage) in univariate analyses. Effect modification with cardiometabolic risk factors was assessed by adding the cross-product term of physical activity and cardiometabolic risk groups to the multivariable model.

## Results

At entry, women with three or four cardiometabolic risk factors, compared to those with no risk factors, were older, have higher BMI, were more likely to be Black, report fair/poor health, to have diabetes, higher blood pressure and report less physical activity (Table 1).

**Table 1:** Baseline metabolic characteristics stratified by physical activity level in 8543 participants with breast cancer in the WHI study

	Physical activity level				
	0 1987 N (%)	> 0–2.9 892 N (%)	3–8.9 1958 N (%)	≥ 9 3706 N (%)	*P* value
*Patient characteristics*					
Age group (enrollment)					
50 to 54	229 (12)	125 (14)	253 (13)	490 (13)	0.4278
55 to 59	440 (22)	201 (23)	431 (22)	785 (21)	
60 to 69	939 (47)	407 (46)	922 (47)	1741 (47)	
70 to 79	379 (19)	159 (18)	352 (18)	690 (19)	
Race/ethnicity					
White	1658 (84)	759 (86)	1713 (89)	3351 (91)	< 0.0001
Black	193 (10)	73 (8)	119 (6)	166 (5)	
Hispanic	63 (3)	27 (3)	44 (2)	63 (2)	
American Indian	6 (0)	5 (1)	8 (0)	11 (0)	
Asian/Pacific Islander	51 (3)	16 (2)	50 (3)	72 (2)	
Education					
High school or less	497 (25)	175 (20)	345 (18)	503 (14)	< 0.0001
> High school/GED	1474 (75)	713 (80)	1596 (82)	3180 (86)	
CT participant					
Observational Arm	967 (49)	434 (49)	1033 (53)	2418 (65)	< 0.0001
Clinical Trial Arm	1020 (51)	458 (51)	925 (47)	1288 (35)	
No mammogram in last 2 years					
Mammogram within 2 years	1582 (82)	749 (86)	1673 (88)	3272 (91)	
No mammogram within 2 years	344 (18)	119 (14)	232 (12)	343 (9)	< 0.0001
Body-mass Index (BMI) kg/m^2^ (kg/m^2^)					
< 25	415 (21)	234 (26)	632 (32)	1601 (44)	< 0.0001
25–< 30	640 (32)	274 (31)	730 (37)	1279 (35)	
30–< 35	478 (24)	206 (23)	366 (19)	552 (15)	
35 +	440 (22)	174 (20)	220 (11)	238 (6)	
*Cardiometabolic abnormalities*					
Waist circumference					
< 88 cm	844 (42)	422 (47)	1144 (58)	2536 (68)	< 0.0001
≥ 88 cm	1143 (58)	470 (53)	814 (42)	1170 (32)	
History of high cholesterol					
No	1704 (86)	762 (85)	1700 (87)	3217 (87)	0.1943
Yes	283 (14)	130 (15)	258 (13)	489 (13)	
History of diabetes					
No	1852 (93)	849 (95)	1861 (95)	3580 (97)	< 0.0001
Yes	135 (7)	43 (5)	97 (5)	126 (3)	
Blood pressure					
Normal	1031 (52)	458 (51)	1138 (58)	2240 (60)	< 0.0001
High	956 (48)	434 (49)	820 (42)	1466 (40)	
Cardiometabolic abnormalities group (baseline)					
0	470 (24)	233 (26)	669 (34)	1494 (40)	< 0.0001
1–2	1348 (68)	584 (65)	1171 (60)	2052 (55)	
3–4	169 (9)	75 (8)	118 (6)	160 (4)	
*Tumor characteristics*					
Tumor size					
< 1 cm	564 (29)	276 (32)	570 (30)	1156 (32)	0.0096
1–< 2 cm	794 (41)	354 (41)	801 (42)	1500 (41)	
≥ 2 cm	568 (29)	233 (27)	537 (28)	960 (26)	
Paget or diffuse	8 (0)	3 (0)	5 (0)	9 (0)	
Morphology—grading					
Well differentiated	465 (25)	244 (29)	539 (30)	1015 (29)	0.0005
Moderately differentiated	847 (46)	367 (44)	804 (44)	1604 (46)	
Poorly differentiated	470 (26)	206 (25)	439 (24)	798 (23)	
Anaplastic	43 (2)	14 (2)	28 (2)	55 (2)	
# lymph nodes examined					
Median (IQR^+^)	4 (2–13)	3 (1–11)	4 (1–11)	4 (1–11)	0.0254
# positive nodes					
0	1322 (67)	616 (69)	1323 (68)	2554 (69)	0.0103
1–3	322 (16)	140 (16)	320 (16)	551 (15)	
≥ 4	135 (7)	44 (5)	105 (5)	181 (5)	
+ nodes NOS	6 (0)	4 (0)	9 (0)	2 (0)	
None examined	200 (10)	88 (10)	201 (10)	413 (11)	
Summary stage (SEER)					
Localized	1482 (75)	691 (77)	1494 (76)	2895 (78)	0.0053
Regional	505 (25)	201 (23)	464 (24)	811 (22)	
Hormone receptor status					
ER or PR+	1614 (86)	748 (88)	1632 (87)	3073 (88)	0.2144
ER and PR−	257 (14)	103 (12)	252 (13)	432 (12)	
HER2/NEU					
Positive	259 (17)	87 (12)	215 (14)	402 (14)	0.0504
Negative	1298 (83)	625 (88)	1357 (86)	2478 (86)	
*Lifestyle factors*					
Smoking status					
Never smoker	964 (49)	471 (53)	1007 (52)	1709 (47)	< 0.0001
Past smoker	826 (42)	354 (40)	824 (42)	1794 (49)	
Current smoker	171 (9)	58 (7)	108 (6)	169 (5)	
Alcohol intake					
Non drinker	253 (13)	106 (12)	191 (10)	239 (6)	< 0.0001
Past drinker	413 (21)	171 (19)	288 (15)	505 (14)	
< 1 drink per month	295 (15)	110 (12)	249 (13)	385 (10)	
< 1 drink per week	400 (20)	179 (20)	419 (21)	763 (21)	
1 to < 7 drinks per week	409 (21)	217 (24)	557 (29)	1160 (31)	
7 + drinks per week	200 (10)	107 (12)	247 (13)	638 (17)	
Cause of death					
Alive	1219 (62)	586 (67)	1383 (72)	2600 (71)	< 0.0001
Breast cancer	190 (10)	75 (9)	156 (8)	259 (7)	
CVD	192 (10)	70 (8)	122 (6)	269 (7)	
Other cancer	121 (6)	42 (5)	93 (5)	189 (5)	
Alzheimer’s/Dementia	51 (3)	23 (3)	61 (3)	98 (3)	
Other	178 (9)	74 (9)	110 (6)	233 (6)	

Higher physical activity levels at entry were inversely associated with high waist circumference, history of diabetes, high blood pressure, and higher cardiometabolic composite scores (all *P* < 0.0001) (Table 2).

**Table 2:** Association between quartiles of activity level and mortality risk in 8543 participants with breast cancer in the WHI study

# of participants		Physical activity level group				P_trend_	HR (95% CI)	P_Interaction_^c^
		0 N = 1987	> 0–2.9 N = 892	3–8.9 N = 1958	≥ 9 N = 3706			
*All-cause mortality*^a^								
All	# Deaths	768	306	575	1106			
	HR (95% CI)	1.00	0.96 (0.84–1.10)	0.80 (0.72–0.90)	0.86 (0.78–0.95)	< 0.001	0.95 (0.92–0.98)	
# of metabolic risk factors								
0	HR (95% CI)	1.00	0.98 (0.71–1.36)	0.83 (0.65–1.07)	0.84 (0.67–1.05)	0.09	0.94 (0.88–1.01)	0.75
1–2	HR (95% CI)	1.00	0.96 (0.82–1.13)	0.76 (0.66–0.87)	0.84 (0.75–0.95)	0.001	0.94 (0.90–0.97)	
3–4	HR (95% CI)	1.00	1.10 (0.73–1.66)	0.87 (0.61–1.26)	0.91 (0.65–1.28)	0.48	0.96 (0.86–1.07)	
*Breast cancer mortality*^b^								
All	# Deaths	190	75	156	259			
	HR (95% CI)	1.00	1.00 (0.76–1.31)	0.92 (0.74–1.15)	0.85 (0.70–1.04)	0.09	0.95 (0.89–1.01)	
# Of metabolic risk factors								
0	HR (95% CI)	1.00	1.16 (0.64–2.09)	0.85 (0.54–1.34)	0.92 (0.62–1.37)	0.54	0.96 (0.85–1.09)	0.46
1–2	HR (95% CI)	1.00	0.86 (0.61–1.20)	0.85 (0.65–1.11)	0.77 (0.60–0.99)	0.04	0.92 (0.85–1.00)	
3–4	HR (95% CI)	1.00	2.71 (0.85–8.64)	0.82 (0.28–2.38)	1.18 (0.45–3.11)	0.87	0.97 (0.72–1.32)	

Median follow-up since enrollment was 19 years, median follow-up since breast cancer diagnosis was 9.5 years

^a^The all cause multivariate model adjusts for age group, race/ethnicity alcohol intake, smoking status, BMI kg/m^2^, # of metabolic risk factors, hormone receptor status, grade, HER2/NEU number of examined lymph nodes, number of positive lymph nodes, tumor size, and stage

^b^The breast cancer multivariate model adjusts for age group, alcohol intake, # of metabolic risk factors, hormone receptor status, grade, # positive lymph nodes, tumor size, and stage

^c^P_Interaction_ was calculated with the cross-product between physical activity level, and # of metabolic risk factors the separate multivariate Cox proportional hazards model

Physical activity levels and cardiometabolic risk factors were determined at study entry. Incident breast cancers were diagnosed 9.5 years (median) from entry and were subsequently additionally followed for 9.5 years (median) after breast cancer diagnosis. In the multivariable models (Table 2), higher physical activity levels were associated with significantly lower all-cause mortality following a breast cancer diagnosis (HR 0.86, 95% CI 0.78–0.95, trend *P* < 0.001) but were not associated with lower breast cancer-specific mortality (HR 0.85, 95% CI 0.70 to 1.04, trend *P* = 0.09). After stratifying by cardiometabolic risk factor groups, the association between higher physical activity at entry and lower all-cause mortality following breast cancer diagnosis was found only in participants with 1–2 metabolic risk factors. There was no statistically significant interaction between cardiometabolic risk factor group and associations of physical activity and breast cancer outcomes.

## Discussion

In prior WHI reports, both higher physical activity levels [13] and fewer cardiometabolic risk factors [4] were associated with lower all-cause mortality following a breast cancer diagnosis in postmenopausal women. While in the current analyses, with longer follow-up, higher physical activity levels continued to be significantly associated with lower all-cause mortality following a breast cancer diagnosis, no significant interaction was seen between physical activity, number of cardiometabolic risk factors and breast cancer mortality. Thus, our study hypothesis was not supported.

To our knowledge, no study has examined the relationship between physical activity, cardiometabolic risk factors, and mortality among women with breast cancer. Current study findings support the established inverse association between physical activity and all-cause mortality among breast cancer survivors [3] as current study participants with breast cancer with higher physical activity levels had lower all-cause mortality. However, current study findings did not demonstrate that cardiometabolic risk factors modified this association.

In contrast, cardiometabolic risk factors modified the effect of a dietary intervention on breast cancer outcomes, as evaluated in the WHI randomized Dietary Modification (DM) trial. In the WHI DM trial, all 48,835 participants were free of prior breast cancer at entry and were randomly assigned to a low-fat dietary-pattern (40%) or a usual diet comparison group (60%). Changes associated with the dietary intervention included reduced fat intake; increased fruit, vegetable, and grain consumption, and weight reduction [15]. While the trial was ongoing, the dietary intervention was also shown to reduce metabolic syndrome components when determined 3 years after entry [14]. After long term, 19.6-year (median) follow-up, compared to women in the comparison group, women in the low-fat dietary intervention group had a statistically significant 21% reduction in death from breast cancer measured from study entry (132 [0.037% annualized risk] v 251 [0.047%] deaths, respectively; HR 0.79 95% CI 0.64–0.97, *P* = 0.02) [15]. In a secondary analysis, women with 3–4 cardiometabolic risk factors, compared to women with no cardiometabolic risk factors, had a significantly higher risk of death from breast cancer, however, those with 3–4 cardiometabolic risk factors randomized to the dietary intervention had a statistically significant 69% reduction in this risk (HR 0.31 95% CI 0.14–0.69, interaction *P* = 0.01, compared to women with 0 or 1–2 risk factors) [12]. Thus, in a randomized clinical trial setting, women having more metabolic syndrome components were more likely to benefit, in terms of reduction in breast cancer mortality, from a lifestyle intervention, randomization to a low-fat dietary pattern.

There is a study which could assess relationships among physical activity, cardiometabolic risk factors, and breast cancer outcome. The ongoing Women's Health Initiative Strong and Healthy (WHISH) pragmatic physical activity intervention trial has completed randomization of 49,331 postmenopausal women testing whether a physical activity intervention reduces major cardiovascular events and all-cause mortality [16]. A secondary analysis of associations among physical activity, metabolic syndrome components and breast cancer mortality would be well-powered to provide definitive assessment.

Current study strengths include the large sample size, prospective study design, use of a previously developed physical activity assessment tool, long follow-up and centralized, adjudicated breast cancer incidence and mortality outcomes. The study has limitations. First, the observational design precludes causal inferences. Second, reliance on questionnaire data for diabetes and cholesterol and reliance on baseline cardiometabolic risk factors and recreational physical activity assessment are limitations as well. Regarding the interval between cardiometabolic risk factor assessment, in a prior analysis in a WHI subgroup, metabolic syndrome status measured earlier in time (more years prior to breast cancer diagnosis) was more predictive of breast cancer risk compared to determinations made closer to breast cancer diagnosis [17, 18].

## Conclusions

In summary, the association of higher physical activity level with lower mortality risk in postmenopausal women with breast cancer did not differ significantly across cardiometabolic risk factor groups. Future studies could consider examining changes in cardiometabolic risk factors and physical activity patterns over time among women with breast cancer.

## Data Availability

The following data will be made available beginning 1 July 2022: the identified participant data and data dictionary (information about data sharing for the Women’s Health Initiative can be found at: www.WHI.orgy/researchers/data/Documents/WHI%20Data%20Sharing%20Statement.pdf. For these analyses, data will be publicly available two years after publication of this article. The following supporting documents are available: statistical/analytical and informed consent form (www.whi.org/researchers/studydoc/SitePages/Protocol%20and%20Consents.aspx).
